# Dengue Fever-Associated Maculopathy and Panuveitis in Australia

**DOI:** 10.1155/2016/5704695

**Published:** 2016-12-19

**Authors:** K. G.-J. Ooi, H. Inglis, N. Paramanathan, J. A. Downie, M. P. Hennessy

**Affiliations:** Department of Ophthalmology, Prince of Wales Hospital, Barker Street, Randwick, NSW 2031, Australia

## Abstract

*Purpose*. To describe a case of dengue fever-associated maculopathy and panuveitis to raise awareness of these ophthalmic complications of dengue in Australia in the light of recent increasing numbers of outbreaks from equatorial through to tropical Australia.* Case Report*. A 37-year-old Caucasian Australian male returning from Cambodia presented with a bilateral dengue fever-associated maculopathy with left panuveitis diagnosed clinically and haematologically. Automated perimetry revealed bilateral paracentral scotomas while optical coherence tomography demonstrated the maculopathies to be of the diffuse retinal thickening type in the right eye and acute macular neuroretinopathy (AMN) type in the left eye. He was treated conservatively with only topical steroids and cycloplegia and made a full clinical visual recovery.* Conclusion*. Our case study underscores the importance of the awareness of the ophthalmic complications of dengue fever as despite their rarity they can be potentially sight threatening. The incidence of these complications is likely to rise in Australia with increased global warming and the distribution of* Aedes aegypti* into subtropical Australia.

## 1. Introduction

Dengue fever is a human arbovirus transmitted through the bite of an infected* Aedes aegypti* or* albopictus *mosquito with clinical presentation ranging from a mild-flu type illness to a life-threatening haemorrhagic fever. Its distribution in tropical and subtropical regions covers over one hundred countries [1]. Ophthalmic complications are uncommon but potentially sight threatening. Our case report aims to raise awareness of these ophthalmic complications of dengue in Australia as although dengue is not naturally endemic in Australia* Aedes aegypti* does exist in northern Queensland where major outbreaks have occurred [2]. Outbreaks can occur when the virus is introduced to the local mosquito population by infected foreign nationals or residents infected while sojourning overseas [2].

## 2. Case Presentation

A healthy 37-year-old Caucasian Australian male presented to medical services in Cambodia with fever, myalgia, arthralgia, and severe headache. He was diagnosed clinically with dengue fever, which was subsequently confirmed on haematological investigations. On day 3 of his illness, he developed sudden onset painless, patchy, clouding of vision (right worse than left), and left eye floaters. This coincided with the nadir of his platelet count at 44 × 10^9^/L (normal > 150 × 10^9^/L). A paucity of other symptoms and signs led to an initial diagnosis of dengue fever-associated retrobulbar optic neuritis, which has been described [3].

**Figure 1:**
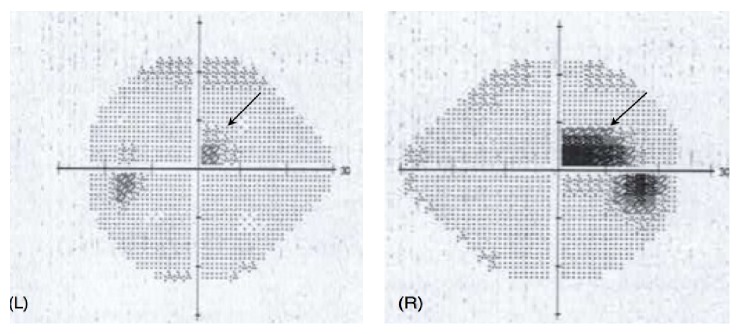
Grey-scale images from Humphrey visual field testing demonstrating paracentral scotomas, right worse (R) than left (L).

**Figure 2:**
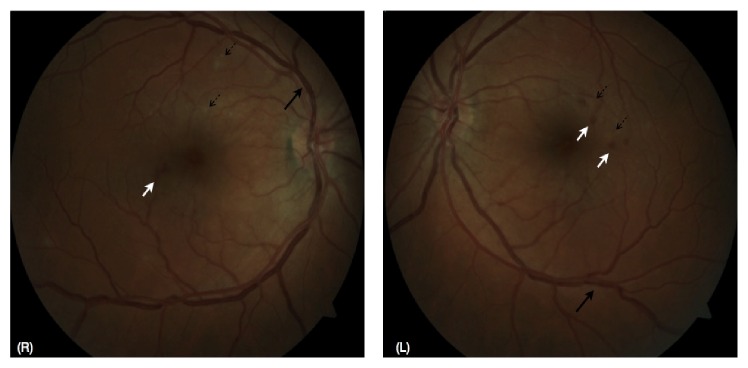
Fundus photographs from the right (R) and left (L) eyes, demonstrating tortuous and beaded retinal veins (heavy black arrows), intraretinal haemorrhages (white arrows), and cotton wool spots (dashed black arrows).

**Figure 3:**
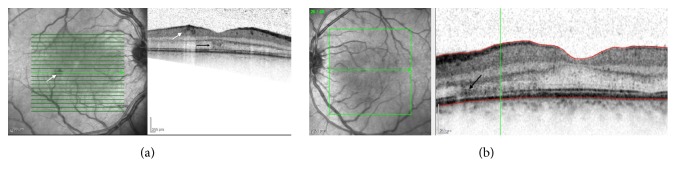
Optical coherence tomographic images of the right (a) and left (b) eyes, demonstrating intraretinal haemorrhage (white arrows (a)), a hyperreflective lesion in the outer nuclear layer with diffuse thickening (black arrow (a)), and increased hyperreflectivity at the level of the outer nuclear layer, external limiting membrane, ellipsoid zone, and interdigitation zone consistent with an acute macular neuroretinopathy (AMN) (black arrow (b)).

He presented for ophthalmic review in Australia 4 days later with symptoms unchanged. An ocular history and systems review for other autoimmune conditions proved noncontributory. His best-corrected visual acuity was 6/6^−1^ (right eye, using eccentric vision) and 6/4 (left eye). Central and paracentral scotomas were noted on Amsler grid testing, worse in the right eye, and confirmed with Humphrey visual field testing (Figure 1). Pupils were equal and reactive to light with no relative afferent pupillary defect. His intraocular pressures were normal. His left eye showed mild ciliary congestion and occasional anterior chamber inflammatory cells with minimal flare but no keratic precipitates. There was mild vitritis with vitreous condensations inferiorly. Neither disc was swollen but there was surrounding hyperaemia and the retinal veins appeared tortuous and beaded. Multiple, perimacular, and intraretinal haemorrhages with occasional cotton wool spots were seen in both eyes (Figure 2). Optical coherence tomography (OCT) was performed which confirmed the fundoscopic findings of intraretinal haemorrhage (Figure 3(a)) and demonstrated hyperreflective lesions involving the outer nuclear layer (Figures 3(a) and 3(b)) and photoreceptor outer segments, encroaching onto the retinal pigment epithelial layer indicating an acute macular neuroretinopathy (AMN) picture as part of a clinical panuveitis complex (Figure 3(b)). The patient declined investigation with fluorescein angiography preventing us from characterising the microangiopathy further. Routine uveitis screening blood tests were noncontributory.

Based on these new posterior pole findings and normal colour vision testing and normal brightness saturation, his diagnosis was changed to dengue fever-associated maculopathy bilaterally with left panuveitis. As his platelet count was recovering, he was managed conservatively with tapering topical steroids and cycloplegia. The anterior chamber inflammatory reaction as well as the vitritis and retinal haemorrhages resolved over the following month as his platelet count returned to normal. He is now fortunately symptom-free with visual acuity recovery to 6/6^−1^ (right eye) and maintained at 6/4 (left eye) best-corrected. Further characterization of his recovery was not able to be performed as he deemed follow-up Amsler grid and visual field testing as well as OCT not necessary.

## 3. Discussion

Although dengue fever is uncommon in Australia, its prevalence is increasing with 1,202 cases of overseas acquired dengue reported in the 2012-2013 season, 1,390 cases in the 2011-2012 season, and 1,133 cases in the 2010-2011 season, on average three times the previous five-year average [4–6]. There were also 212 cases of dengue acquired in Australia [4]. Our case study aims to bring attention to the uncommon but potentially sight threatening ophthalmic complications of dengue fever so that patients are referred to ophthalmology services where appropriate and early in the disease process. To the best of our knowledge this is only the second documented case of an ocular complication associated with dengue fever in Australia [7].

Reported ophthalmic complications of dengue fever include anterior uveitis, retinal vasculitis and oedema, exudative retinal detachment, optic neuritis, ischaemic optic neuropathy, branch retinal artery occlusion, and retinal pigment epithelial disturbance [3, 8, 9]. The wide variety of ocular complications suggests several different pathophysiological mechanisms at play. The most apparent is the viral-induced thrombocytopenia leading to a generalised increased risk of haemorrhage. This accounts for the characteristic clinical features of dengue, specifically petechiae, epistaxis, and bleeding gums and also leads to retinal haemorrhages, both peripherally and at the macula [3, 8]. This is matched by the observation that visual symptoms typically occur in conjunction with the nadir of platelet count [8], as in our patient.

The occurrence of complications such as uveitis, vasculitis, and macular oedema is more suggestive of a different, immune-mediated hyperpermeability, and inflammatory mechanism, supported by the typical presentation of ophthalmic symptoms in the convalescent stage about 1 week after onset of fever [3]. It is known that interactions between the virus, the immune response, and vascular endothelium lead to acute plasma leakage [10]. In the eye this would lead firstly to subretinal fluid and macular oedema with secondary inflammatory processes leading to, for example, the panuveitis in the left eye of our patient as a result of the breakdown of the blood-retina-barrier [8]. Most commonly dengue affects the posterior segment of the eye although Gupta et al. presented a case series of 6 patients where anterior uveitis was accompanied by posterior uveitis in only one patient [11]. Interestingly this cohort presented with symptoms only 3–5 months after dengue fever contraction suggesting a different delayed autoimmune mechanism in these patients. The right eye hyperreflective lesion in Figure 3 could be consistent with the diffuse retinal thickening gradation of maculopathy as defined by Teoh et al. [12]. The left eye maculopathy as demonstrated in Figure 3(b) has been recently further characterised as an acute macular neuroretinopathy (AMN) not only in dengue fever but also classically in association with leukaemia and ulcerative colitis and in a range of more recently described aetiologies [13]. It has been suggested that the thrombocytopenia leads to localisation of the ischaemia at the level of the intermediate capillary plexus in AMN [13].

As a result of the immune-mediated hypothesis, both topical and systemic steroid immunosuppression have been trialled in patients with visual loss [3, 8]. Larger studies are required, however, to determine the effectiveness of this treatment. This intervention may be warranted in cases where posterior pole inflammation has led to unremitting, severe, and central visual loss. Persistent scotomas are most likely in cases with foveolitis and less likely in those with diffuse retinal thickening only [12]. However, the use of systemic steroids in acute viral infection may worsen the general condition and should only be considered in a multidisciplinary context with both ophthalmic and infectious disease physician input. An alternative may be local intraocular or subconjunctival steroid injection in cases of severe visual deterioration. As the scotomas were limited and visual acuity was still good in our patient, oral steroids were not used. This is in contrast to the patient of Gupta et al., in their previously mentioned case series, with severe vitritis where oral steroids were used [11]. In this scenario of late-onset uveitis, steroid-worsening of the viral infection may potentially be less of a concern.

The ophthalmic complications of dengue fever are uncommon but not rare, with one series reporting a rate of 10% of maculopathy in hospital patients with dengue fever [14]. Ocular complications may be more prevalent than thought as those with pathology in the peripheral retina are less likely to be symptomatic. It is important to note that while many cases are like ours and are fortunately self-limiting, sight threatening complications have been reported to occur in as many as 5–8% of all cases of dengue fever [13]. Scotomas may persist for months or longer despite normalisation of visual acuity on the Snellen chart which may have a significant impact on patient functioning [8]. Persistent scotomas in AMN have been documented to persist over a 6-month period and related to irreversible thinning of the outer nuclear layer and incomplete restoration of the interdigitation zone [15, 16]. This may have been the case in our patient's right eye but his forgoing of follow-up investigations precluded the detection of any residual visual disturbance. Additionally, major ophthalmic complications such as retinal artery occlusion can occur without severe systemic manifestations of dengue fever [9]. Given the recent outbreaks in Australia and that these trends are likely to continue with the increase in global warming and urban development [17, 18], more ophthalmic presentations are likely to occur even into subtropical Australia where* Aedes aegypti* is prevalent [19].

In summary, in cases of dengue fever such as ours with visual deterioration, consideration of use of steroid immunosuppression is warranted in the hopes of preventing further deterioration and eventual visual recovery. The maculopathy present in our patient's right eye is consistent with AMN which has been further characterised in dengue fever only recently. Australian eye health care practitioners should be aware that dengue fever is encountered in our country and at increasing rates. They should also be aware of the potential ophthalmic complications and that a published framework exists for the surveillance, prevention, and control of dengue virus in Australia [19].
